# CXCL16/CXCR6 Axis Drives Microglia/Macrophages Phenotype in Physiological Conditions and Plays a Crucial Role in Glioma

**DOI:** 10.3389/fimmu.2018.02750

**Published:** 2018-11-27

**Authors:** Francesca Lepore, Giuseppina D'Alessandro, Fabrizio Antonangeli, Antonio Santoro, Vincenzo Esposito, Cristina Limatola, Flavia Trettel

**Affiliations:** ^1^Department of Physiology and Pharmacology, Sapienza University, Rome, Italy; ^2^IRCCS Neuromed, Pozzilli, Italy; ^3^Department of Molecular Medicine, Sapienza University, Laboratory affiliated to Istituto Pasteur Italia–Fondazione Cenci Bolognetti Rome, Italy; ^4^Department of Neurology and Psychiatry, Sapienza University, Rome, Italy; ^5^Department of Physiology and Pharmacology, Sapienza University, Laboratory affiliated to Istituto Pasteur Italia-Fondazione Cenci Bolognetti Rome, Italy

**Keywords:** CXCL16, CXCR6, tumor microenvironment, glioma, microglia, neuroinflammation

## Abstract

Microglia are patrolling cells that sense changes in the brain microenvironment and respond acquiring distinct phenotypes that can be either beneficial or detrimental for brain homeostasis. Anti-inflammatory microglia release soluble factors that might promote brain repair; however, in glioma, anti-inflammatory microglia dampen immune response and promote a brain microenvironment that foster tumor growth and invasion. The chemokine CXCL16 is expressed in the brain, where it is neuroprotective against brain ischemia, and it has been found to be over-expressed in glioblastoma (GBM). Considering that CXCL16 specific receptor CXCR6 is diffusely expressed in the brain including in microglia cells, we wanted to investigate the role of CXCL16 in the modulation of microglia cell activity and phenotype, and in the progression of glioma. Here we report that CXCL16 drives microglia polarization toward an anti-inflammatory phenotype, also restraining microglia polarization toward an inflammatory phenotype upon LPS and IFNγ stimulation. In the context of glioma, we demonstrate that CXCL16 released by tumor cells is determinant in promoting glioma associated microglia/macrophages (GAMs) modulation toward an anti-inflammatory/pro-tumor phenotype, and that *cxcr6ko* mice, orthotopically implanted into the brain with GL261 glioma cells,survive longer compared to wild-type mice. We also describe that CXCL16/CXCR6 signaling acts directly on mouse glioma cells, as well as human primary GBM cells, promoting tumor cell growth, migration and invasion. All together these data suggest that CXCL16 signaling could represent a good target to modulate microglia phenotype in order to restrain inflammation or to limit glioma progression.

## Introduction

Modification of local brain microenvironment can be sensed by microglia cells, which respond to preserve brain homeostasis, or to exacerbate brain damage. Understanding the mechanisms of microglia communication in the brain is important to identify molecular players that can be used as targets to counteract brain damage and preserve brain homeostasis. Within the brain, microglia are plastic cells that constantly monitor brain parenchyma to sense local perturbation and, depending on specific environmental cues, can change their phenotype and functional activity promoting inflammatory or anti-inflammatory conditions (1, 2).

While chemokines were originally discovered for their ability to regulate leukocyte trafficking, it is now accepted that, beyond chemotaxis, these molecules exert pleiotropic activities in the context of brain physiology, as well as brain cancer (3–8). A crucial role for chemokines and their receptors as mediators of homeostatic crosstalk between neurons and glia has emerged (9, 10) and we have recently shown that the trans-membrane chemokine CXCL16, through its unique receptor CXCR6, orchestrates cell cross-talk to promote neuroprotection against glutamate-induced excitotoxic insults (11); to mediate endogenous protective mechanisms to counteract neuronal damage during brain ischemia (12); and to modulate neurotransmitter release in the hippocampus (13).

Glioblastoma (GBM) is a high grade tumor with a poor prognosis. Despite aggressive surgical resection and chemotherapy, GBM patients undergo tumor recurrence due to the highly infiltrative nature of the tumor cells, and to the persistence of chemotherapy-resistant cells (14). Glioma cells release molecular regulators, such as cytokines and growth factors, which may act in autocrine ways promoting tumor cell proliferation and invasion or in paracrine ways contributing to the establishment of a pro-tumor-microenvironment (15–17). Non-tumor cells of the brain parenchyma, such as astrocytes, endothelial cells, but also microglia, as well as infiltrating peripheral immune cells, sense glioma, and contribute to the formation of a tumor niche that provides a crucial environment for glioma progression. In this context, the cross-talk between tumor cells and glioma associated microglia/macrophages (GAMs) leads to GAMs polarization toward an anti-inflammatory, immunosuppressive, pro-invasive phenotype that support tumor growth and invasion (18).

GBM cells express chemokines that regulate tumor cell proliferation, invasion, angiogenesis, as well as the maintenance of an immunosuppressed microenvironment (19, 20). CXCL16 is expressed in human glioma (21), while the presence of CXCR6 is controversial, likely associated with glioma-stem cells (21, 22).

In the present paper we highlight for the first time a major role of CXCL16/CXCR6 axis in driving microglia polarization toward an anti-inflammatory phenotype that: in inflammatory context provides a neuroprotective mechanism to limit brain damage; in the context of glioma triggers a pro-tumoral microenvironment. Moreover, we show that CXCL16 produced by glioma cells directly stimulates the CXCR6 expressed by tumor cells, promoting their proliferation, migration and invasion.

## Materials and methods

### Materials

Recombinant murine CXCL16 (cat#250-28) and CXCL12 (cat#250-20A) were from Peprotech; IL-4 (cat#12340045) and IFNγ (cat#12343536) were from Immunotools; anti-CXCL16 (cat# MAB503-100), mouse CXCR6 PE-conjugated antibody (cat# FAB2145P−025, RRID:AB_2089531), human CXCR6 PE-conjugated antibody (cat#FAB699P−025, RRID:AB_2261441) were from R&D System, APC anti mouse H-2Kb/H-2Db (cat#114613) and APC anti mouse CD1d antibody (cat#123521, RRID:AB_2715919) were from Biolegend; APC rat anti-mouse CD44 (cat# 559250), PE rat anti-mouse CD274 (PD-L1)(cat#558091) were from BD Pharmingen; IgG from rat serum antibody (cat#l4131, RRID: AB_1163627), LPS (cat#L4391), 2′,7′-Dichlorofluorescin diacetate (cat#D6883) were from Sigma-Aldrich; anti-Iba1antibody was from Wako (cat#019-19741, RRID:AB_839504); anti-GFAP (cat#NB300-141, RRID:AB_10001722), anti-5-bromo-2-deoxyuridine (cat#NB500169, RRID:AB_341913) antibodies were from Novus Biological and anti-Arg1antibody was from Santa Cruz (cat#sc-271430 RRID:AB_10648473); anti-CD68 antibody (cat#MCA1957T, RRID:AB_322219) was from AbD Serotec;. Secondary Abs were from DAKO; Microbeads CD11b^+^ were from Miltenyi Biotec; Trans-well inserts were from BD Labware (cat#353097); IPTG (Dioxane-free) was from Thermo Fisher (cat#AM9464). Hematoxylin, eosin, and BSA were from Sigma-Aldrich. All cell culture media, fetal bovine serum (FBS), goat serum, penicillin G, streptomycin, glutamine, the Thermo Script RT-PCR System, and Hoechst (cat#33342, RRID:AB_10626776) were from Invitrogen. 5-Bromo-2′-Deoxyuridine (BrdU) (cat#B5002) and lentiviral shRNA clones targeting murine CXCR6 and CXCL16 were from Sigma-Aldrich. Elisa kit for Interleukin 1 Beta (IL-1β) was from Claude-Clone Corp. (cat#SEA563Mu); Elisa kit for CXCL16was from RayBiotech (cat#ELMCXCL16); Griess reagent kit for Nitrite determination was from Molecular Probe (cat#G-7921), Red fluorescent FluoSpheres (0.03%) were from Invitrogen.

### Ethics statement

This study was approved: by the Institutional Review Board of the Policlinico Umberto I Medical Center according to the Bioethics and Safety Act and the Declaration of Helsinki. Each participant provided oral informed consent (according to the principle 22 of Ethical Principles for Medical Research Involving Human Subjects); by the Institutional Review Board of Neuromed Medical Center according to the Bioethics and Safety Act and the Declaration of Helsinki. Each participant provided written informed consent.

### Human tissue samples

Tumor specimens (GBM 1, 2, 3, 11, 13, 14, 19, 28, 40, 45, 46, 51, 58) were obtained at Policlinico Umberto I (Rome) and Neuromed (Pozzilli, Isernia) from adult glioblastoma (GBM). Within half an hour from surgical resection GBM tissues were processed to obtain primary GBM cells or frozen for molecular study. Histopathological typing and tumor grading were done according to the WHO criteria resulting as grade IV. Normal cerebral tissues derived from the prefrontal cortex of patients who died from heart failure were kindly provided by Dr. Eleonora Aronica, with ethics approval of Amsterdam University.

### Animals and cell cultures

The experiments described in the present work, were approved by the Italian Ministry of Health in accordance with the guidelines on the ethical use of animals from the European Community Council Directive of September 22, 2010 (2010/63/EU). Wild type mice C57BL/6J (cat# JAX: 000664, RRID: IMSR_JAX:000664) and Homozygous cxcr6gfp/gfp knock-in mice (cat# JAX: 005693, RRID: IMSR_JAX:00569) (23), in which the coding region of CXCR6 receptor has been substituted with the coding region of the green fluorescent protein, were from Jackson Laboratory. In the present manuscript, we refer to cxcr6gfp/gfp knock-in mice as *cxcr6ko* mice, and to C57BL/6J as *wt* mice.

The mouse GL261 glioma cell line (RRID:CVCL_Y003; kindly provided by Dr. Serena Pellegatta, Istituto Di Ricovero e Cura a Carattere Scientifico, Besta, Milan) was cultured in growth medium (DMEM with 20% heat-inactivated FBS, 100 IU/ml penicillin G, 100 μg/ml streptomycin, 2.5 μg/ml amphotericin B, 2 mM glutamine, and 1 mM sodium pyruvate). GL261/CD133^+^ cells were obtained as previously described in Garofalo et al. (24). The cell lines were tested for mycoplasma contamination (negative). Primary GBM cells were obtained as previously described (25). Briefly tumor tissues were mechanically dissociated to cell suspensions and red blood cells were lysed with hypotonic buffer. Tumor cells were re-suspended in serum-free growth medium and cultured at 37°C in humidified atmosphere with 5% CO2. Twenty-four hours later, non-adherent cells were removed and the growth medium was supplemented with 10% heat-inactivated FBS. Cells were sub-cultured when confluent. In the current study, primary GBM cells, were used within 1–3 passages, and were named GBM13, GBM19, GBM40, and GBM45.

### Microglia culture and polarization

Microglia cells were obtained from mixed glia cultures derived from the cerebral cortices of post-natal day 0–2 (p0–p2) *wt* mice. Cortices were chopped and digested in 15 U/ml papain for 20 min at 37°C. Cell suspensions were plated (5 × 10^5^ cells/cm^2^) on poly-L-lysine (0.1 mg/ml) coated flasks in growth medium supplemented with 10% FBS. After 9–11 days, cultures were shaken for 2 h at 37°C to detach and collect microglia cells. These procedures gave almost pure microglial cell populations as previously described (26). For microglia polarization, cells were seeded on poly-L-lysine (cat#P2636 from Sigma-Aldrich) coated six-well plate and the day after they were treated with LPS 100 ng/ml + IFNγ 20 ng/ml or glioma conditioned medium (GCM) with rat AbCXCL16 or IgG (1 μg/ml) for 24 h.

### CXCR6 and CXCL16 silencing by shRNA interference

GL261 cells were transduced by lentiviral particles directing IPTG-inducible expression of CXCR6 shRNA or constitutive expression of CXCL16 shRNA constructs. Cells (1.6 × 10^4^) were plated in 96-well plates and infected for 24 h according to the manufacturer's instructions. Transduced cells were selected with 2 μg/ml puromycin for 3–12 days. IPTG (5 mM) was added for 10 days to culture medium to induce CXCR6 shRNA expression. Knockdown efficiency of CXCR6 receptor and CXCL16 was evaluated by PCR or chemotaxis assay. Silenced cell lines were named GL261shCXCR6 and GL261shCXCL16 in this study.

### Chemotaxis and invasion *in vitro* assays

GL261, GL261shCXCR6 and human primary GBM cells were pre-incubated in chemotaxis medium (DMEM without glutamine, 100 IU/ml penicillin G, 100 μg/ml streptomycin, 0.1% BSA, and 25 mM HEPES, pH 7.4) with AraC (10 μM, 15 min) to block cell duplication. Cells (4 × 10^4^) were plated in the upper wells of 48-well boyden chamber (NeuroProbe) on 8 μm-pored Poly-L-Lysine coated membrane. The lower wells contained CXCL16 (0.1, 1, 10, 50, or 100 nM), CXCL12 (50 ng/ml), or vehicle (C). Cells were left migrate for 4 h (GBM cells) or 24 h (GL261). For invasion assay, GL261 and GBM19 were plated at a density of 2 × 10^4^ cells/cm^2^ on matrigel-coated transwells (8 μm pored membrane) and left invade toward CXCL16 (1, 10 nM) or vehicle, respectively, for 48 or 24 h at 37°C. Migrated/invaded cells were fixed and stained with a solution containing 50% isopropanol, 1% formic acid, and 0.5% (w/v) brilliant blue R 250. For each membrane, stained cells were counted in at least 20 fields with a 32 × objective of a phase-contrast microscope (Zeiss).

### MTT assay

GL261, GL261shCXCR6, and GBM19 cells were seeded into 96 well plates (5 × 10^3^) and treated with vehicle (C) or with CXCL16 (10 nM) for different time points (0, 24, 48, 72, or 96 h). MTT solution (500 μg/ml) was added into each well for 1.5 h. DMSO was then added to stop the reaction and the formazan produced was measured at 570 nm. Viability of cells was expressed relative to absorbance values.

### Western-blot

For protein analysis, microglial cells (6 × 10^5^) were seeded on six-well plates and treated with vehicle, CXCL16 (200 nM), glioma conditioned medium (GCM) with or without rat AbCXCL16 for 24 h; cells were washed with PBS and lysed in hot 2 × Laemmli buffer, boiled 5 min, and sonicated. The same amount of proteins was separated on 12% SDS-polyacrylamide gel and analyzed by Western immunoblot using the following primary antibodies: ARG-1 1:200, ACTIN 1:2,000. HRP-tagged goat anti-mouse and anti-rabbit-IgG were used as secondary antibodies (1:2,000; Dako), and detection was performed by the chemiluminescent assay Immun-Star WesternC Kit (Bio-Rad, CA). Densitometric analysis has been carried out with Quantity One software (Biorad, CA).

### Phagocytosis assay

Microglial cells were seeded on poly-L-lysine-treated 10 mm glass coverslips (7 × 10^4^ cells) and stimulated with CXCL16 (200 nM) or vehicle for 24 h and GCM with or without rat AbCXCL16 for 24 h. Medium was then removed, 0.05% (corresponding to 1.8 × 10^7^ spheres/ml) red fluorescent FluoSpheres were added for 1 h in serum-free medium (0.1% BSA), and nuclei were stained by Hoechst. Cells were washed three times with PBS to remove non-phagocytized spheres and fixed in 4% PFA for 1 min. Phagocytosis was quantified by counting the number of phagocytizing cells (scoring as positive only cells with at least five FluoSpheres to avoid possible false positives due to sphere adhesion to cell surface) in at least 20 random fields per coverslip.

### Form factor calculation

Microglia were seeded on glass coverslips, treated as necessary, fixed, permeabilized, blocked and stained with Alexa-Fluor 488 Phalloidin (Invitrogen) for 20 min together with Hoechst. Fluorescent images were processed using the MetaMorph 7.6.5.0 software (Molecular Device, Sunnyvale, CA, USA), and form factor was calculated according the formula: 4π area/perimeter^2^ (27). Form factor is a parameter taken as 1 for round cells, and correspondingly < 1 when the morphology deviates from the spherical shape.

### Nitric oxide (NO) measurement

NO production by microglia cultures was assessed by measuring nitrite accumulation in the culture medium by Griess Reagent Kit according to manufacturer's instructions (Molecular Probes, MA, USA). The absorbance was measured at 570 nm in a spectrophotometer microplate reader (BioTek Instruments Inc., VT, USA).

### ELISA assay

Microglial cells (6 × 10^5^ cells) were seeded onto a 6-well culture plate, after 24 h cells were stimulated with LPS 100 ng/ml + IFNγ 20 ng/ml or LPS 100 ng/ml + IFNγ 20 ng/ml + CXCL16 (200 nM) for 24 h. Medium was than collected, centrifuged at 1,000 × g for 20 min, and supernatant was stored at −80°C. Control cells were stimulated only with vehicle. IL-1β present in the supernatant was measured using a specific ELISA for mouse IL-1β (Cloud-Clone Corp.) as described by the manufacturer. For each sample, cells were detached and proteins were quantified (BCA assay). For quantification of mouse CXCL16 in glioma conditioned medium (GCM) we used the mouse CXCL16 ELISA Kit (RayBiotech, Norcross, GA, USA) as described by the manufacturer. All supernatants were centrifuged (1,000 × g for 5 min) to eliminate floating cells and then samples were 10-fold concentrated with 10 KDa Microcon Centrifugal Filter devices (Merck Millipore, Darmstadt, Germany). Samples were measured in duplicate and confirmed in two independent experiments.

### Reactive oxygen species (ROS) measurement

Primary microglia cultures (3 × 10^5^ cells) were treated for 18 h with 200 nM CXCL16 and then cells were incubated with 20 μM of 2′,7′-Dichlorodihydrofluorescein diacetate (DCF-DA, Sigma-Aldrich, #D6883) for 30 min at 37°C. Cell fluorescence was detected in FL1 channel and analyzed with a FACSCanto II (BD Biosciences). Data were elaborated using FlowJo v9.3.2 software (TreeStar, Ashland, OR, USA).

### Cytofluorimetric analysis

Cells were harvested in PBS with 5 mM EDTA and washed in staining buffer (PBS without Ca^2^+ Mg^2+^, 0.5% BSA, 2 mM EDTA, 0.025% NaN_3_). mAbs directly conjugated to PE and APC fluorochromes and specific for the following antigens were used: MHC class I (APC anti-mouse H-2Kb/H-2Db, BioLegend), CD1 (APC anti-mouse CD1d, clone 1B1, BioLegend), CD44(APC rat anti-mouse CD44, clone IM7, BD Pharmingen), PD-L1 (BD Pharmingen), CXCR6 (R & D systems). Corresponding isotypes were used for negative control. Immunostaining was performed with saturating amounts of Abs for 30 min at 4°C. Samples were acquired with a flow cytometer FACSCanto II (BD Biosciences) and data were elaborated using FlowJo 9.3.2 software (TreeStar).

### Reverse transcript PCR (RT-PCR) and quantitative real time PCR (RT-qPCR)

Samples were lysed in TRYzol reagent for isolation of total RNA. The quality and yield of RNAs were verified using NANODROP One (Thermo Scientific). For RT-PCR one microgram of total RNA was reverse transcribed using ThermoScript RT-PCR System and 150 ng of the reverse transcription products were used as a template for PCR amplification. The PCR protocol was as follows: 95°C for 5′, 30 cycles 94°C for 30″, 55°C for 30″, and 72°C for 30″. MJ Mini Thermal Cycler (Bio-Rad) was used for all reactions and amplification products were analyzed on 1.8% agarose gel stained with ethidium bromide. For RT-qPCR Reverse transcription reaction was performed in a thermocycler using IScript TM RT Supermix (Biorad) under the following conditions: incubation, 25°C, 5′; reverse transcription, 42°C, 45′; inactivation, 85°C, 5′. Real Time-PCR was carried out in a I-Cycler IQ Multicolor RT-PCR Detection System using Sso Fast Eva Green Supermix (Biorad). The PCR protocol consisted of 40 cycles at 95°C, 30″ and 60°C, 30″. For quantification analysis, the comparative Threshold Cycle (Ct) method was used. The Ct values from each gene were normalized to the Ct value of GAPDH in the same cDNA samples. Relative quantification was performed using the 2^−ΔΔ*Ct*^ Ct method (28) and expressed as fold increase in arbitrary values. Primers sequences are reported in Supplementary Table 1. Primers used for CXCR6 and CXCL16 were not intron spanning, and “no-RT” reactions were used as controls to rule out priming off of genomics DNA. As control for *cxcl16* and *cxcr6* mRNA expression, we used RNA from Human fibroblast cell line HFF-1 (ATCC® SCRC-1041™, RRID:CVCL_3285); RNAs from Mouse fibroblast NIH/3T3 cells (ATCC® CRL-1658™, RRID:CVCL_0594); RNA from primary human T lymphocytes kindly provided by Dr. Samantha Cialfi, Department of Molecular Medicine, Sapienza, Rome); RNA from mouse primary CD4^+^ T cells derived from spleen.

### Brain injection of glioma cells and survival analysis

Eight week old male mice (*wt* or *cxcr6ko*) were anesthetized with chloral hydrate (400 mg/kg, i.p.) and placed in a stereotaxic head frame. Animals were injected with 1 × 10^5^ GL261, GL261shCXCR6, or GL261shCXCL16 cells at 2 mm lateral and 1 mm anterior to the bregma in the right striatum. Cell suspensions, in sterile phosphate-buffered saline (PBS) (5 μl) were injected with a Hamilton syringe at a rate of 1 μl/min at 3 mm depth. For GL261 shCXCR6, after 10 days of IPTG treatment, cells were injected in mice and shRNA expression was maintained by adding IPTG (10 mM) in drinking water. For survival analysis glioma injected mice were daily monitored. The end points were determined by lack of physical activity or 20% weight loss in glioma-bearing mice. The mean survival time was calculated using the Kaplan–Meier method and statistical analysis was performed using a log-rank test.

### Tumor volume evaluation and brain sections immunostaining

Seventeen days after tumor cell injection glioma-bearing mice were killed and brains were isolated and fixed in 4% buffered paraformaldehyde. Brains were snap frozen and cut in 20 μm coronal brain cryosections. Tumor volume was evaluated with hematoxylin–eosin staining as previously described. Briefly, after staining, brain slices (20 μm of thickness) were analyzed by the Image Tool 3.0 software (University of Texas, Health Science Center, San Antonio, TX, USA). Tumor volume was calculated according to the formula (volume = t × ΣA), where A = tumor area/slice and t = thickness (29). For tumor cell proliferation *in vivo*, 17 days after tumor cells injection glioma-bearing mice were injected intraperitoneally with bromo-2-deoxyuridine (50 mg/kg). Two hours later, mice were killed and brains processed for BrdU immunostaining. For immunostaing analysis cryosections were washed in PBS and blocked with blocking solution (3% goat serum, 0.3% Triton X-100 in PBS) for 1 h at room temperature. Sections were then incubated with specific antibodies (anti-Iba1 1:500, anti-CD68 1:200, anti-GFAP 1:750, anti-BrdU 1:200) in 1% goat serum and 0.1% Triton X-100/PBS solution overnight at 4°C. After several washes, sections were stained with the respective secondary fluorophore-conjugated antibody and Hoechst for nuclei visualization. For Iba1 staining, citrate buffer antigen retrieval protocol was used. For BrdU immunostaining, sections were pretreated with HCl 1N for 15 min, HCl 2N for 25 min at 37°C, and neutralized with 0.1 M borate buffer. Digitized fluorescent cell images were collected using an inverted fluorescence microscope (Nikon Ti Eclipse) and analyzed with MetaMorph analysis software (Molecular Devices, USA).

### BrdU cell immunostaining

GL261 cells were grown on glass coverslips at a density of 5 × 10^4^ cells/cm^2^ and treated for 4 h with CXCL16 10 nM or vehicle. Cells were then incubated with 10 μg/ml BrdU for 30 min, washed with PBS and fixed in 4% paraformaldehyde for 20 min. Fixed cells underwent immunostaining protocols as described for brain sections. Hoechst was used for nuclear staining. BrdU positive cells were counted out of 800 cells for condition.

### Invasion *in vivo* assay

Seventeen days after GL261 injection, mice brains were isolated and fixed in 4% buffered formaldehyde for morphological evaluation. Coronal brain sections (20 μm), prepared using the standard procedures, were stained with hematoxylin and eosin. For analysis of tumor invasiveness, glioma cells protruding more than 150 μm from the main tumor mass were counted in at least 20 fields, obtained from six slices per mice.

### Isolation of CD11b^+^ cells

Glioma-bearing *wt* or *cxcr6ko* mice after 17 days from inoculation were deeply anesthetized and intracardially perfused with ice cold PBS. Brains were removed, cut into small pieces and single-cell suspensions were achieved by enzymatic digestion in trypsin (0.25 mg/ml) solution in Hank's balanced salt solution (HBSS). Cell suspensions were labeled with CD11b^+^ Microbeads, loaded onto a MACS Column (MiltenyiBiotec) and placed in the magnetic field of a MACS Separator. After removing the magnetic field, CD11b^+^ cells were eluted and used for RNA extraction. CD11b^+^ cells were also isolated from human GBM tissues surgically removed from patients as described above.

### Statistical data analysis

Data are expressed as the mean ± SEM. Statistical significance was assessed by Student's *t*-test, Student's paired *t*-test or one-way ANOVA, as indicated; Holm–Sidak, Turkey *post-hoc* test or Student-Newman-Keuls Method were used as a *post-hoc* test. For Kaplan–Meier analysis of survival, the log-rank test was used. All statistical analyses were done using Sigma Plot 11.0 software.

## Results

### CXCL16 drives microglia polarization *in vitro*

Since we have shown that CXCL16 is neuroprotective in ischemia (11, 12), and neuroinflammation plays a role in brain damage following ischemic insult (30, 31), we considered the possibility that CXCL16, acting on CXCR6 expressed by microglia cells (11), might provide protective effects also modulating microglia phenotype.

**Figure 1:**
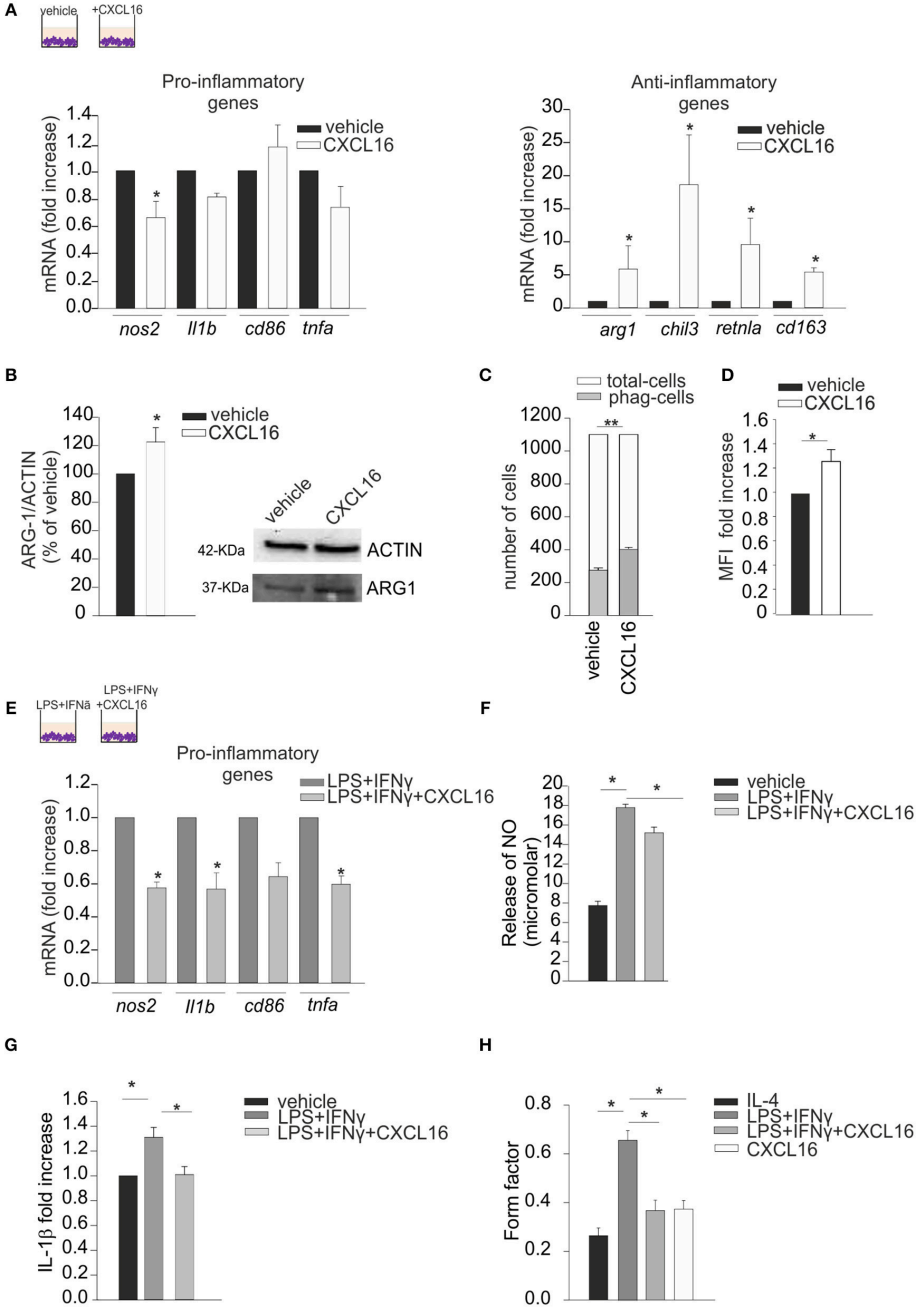
Effects of CXCL16 in modulating microglia phenotype. Expression analysis by RT-qPCR for mRNAs of pro-inflammatory (*nos2, il1b, cd86, tnfa*) or anti-inflammatory (a*rg1, chil3, retnla, cd163*) related genes in primary *wt* microglia treated with: **(A)** vehicle or CXCL16 (200 nM); **(E)** LPS+IFNγ (pro-inflammatory stimulus) in the absence or presence of CXCL16. For each gene data are expressed as specific mRNA fold increase in CXCL16 treated cells normalized to specific mRNA expression in vehicle **(A)**, or in LPS + IFNγ treated cells **(D)**; **(B)** Western-blot analysis of ARG-1 protein expression in microglia cells incubated vehicle or CXCL16. Right, representative image; left histogram bar of the quantification of ARG-1 expression (data are expressed as ARG-1 signal normalized to ACTIN signal). **(C)** Phagocytosis of fluorescent beads in microglia cells stimulated with CXCL16 (200 nM, 24 h), or not (vehicle). Data are expressed as number of cells containing 5 or more beads (gray bars) within total counted cells (white bars); **(D)** ROS production of microglia cells after CXCL16 treatment as evaluated by using the DCF probe. DCF was analyzed as median fluorescence intensity (MFI) by flow cytometry; **(F,G)** Release of NO and IL-1β by microglia cells not stimulated (vehicle) or stimulated with LPS + IFNγ in the absence or presence of CXCL16; for IL-1β data are expresses as fold increase vs. vehicle; **(H)** Form factor analysis of microglia cells treated with IL-4 (anti-inflammatory stimulus), LPS + IFNγ, LPS + IFNγ + CXCL16, or CXCL16. Statistical analysis: Data are expressed as the mean (± s.e.m.) **(A)** *n* = 4, *p < 0.05, Student's *t-*test; **(E)** *n* = 5, ^*^p < 0.05, Student's *t-*test. For each gene, variability in its expression among control conditions in different experiments never exceeded 10%. **(B)** *n* = 4, *p < 0.05, Student's *t*-test; **(C)** *n* = 3, **p < 0.001, Student's *t*-test; **(D)** *n* = 3 experiments in duplicates, *p < 0.05, Student's *t-*test; **(F)** *n* = 7, *p < 0.05, one-way ANOVA followed by Holm–Sidak *post-hoc* test; **(G)** n = 4, *p < 0.05, one-way ANOVA followed by Holm–Sidak *post-hoc* test; **(H)** *n* = 30 cells,*p < 0.05, one-way ANOVA followed by Tukey *post-hoc* test.

We performed *in vitro* experiments treating primary mouse microglia for 24 h with CXCL16 (200 nM) and analyzing the expression of pro- (*nos2, il1b, cd86, tnfa*) and anti- (a*rg1, chil3, retnla, cd163*) inflammatory genes (32) by RT-qPCR: as reported in Figure 1A, CXCL16 increases the expression of anti-inflammatory genes (right panel; *n* = 5 *p* < 0.05; Student's *t*-test), while no significant modulation of pro-inflammatory genes is observed, with the exception of *nos2* (left panel; *n* = 5 *p* < 0.05; Student's *t*-test).The ability of CXCL16 to induce anti-inflammatory polarization was further supported by an increase in ARG-1 protein expression in microglia treated with CXCL16 (200 nM, 24 h) vs. not treated cells (*n* = 4, *p* < 0.05; Student's *t*-test), Figure 1B. Moreover, CXCL16 increases: the number of phagocytizing microglia (measured as number of cells that phagocytized five or more fluorescence beads) (*n* = 3, *p* < 0.001; Student's *t*-test) vs. control, Figure 1C; the production of reactive oxygen species (measured as generated DCF fluorescence) vs. vehicle (*n* = 3 experiments in duplicates, *p* < 0.05; Student's *t*-test), Figure 1D.

We then wanted to verify the hypothesis that CXCL16 could also modulate microglia polarization in the context of pro-inflammatory conditions (LPS, 100 ng/ml + IFNγ, 20 ng/ml, 24 h): as reported in Figure 1E, the presence of CXCL16 (200 nM) significantly reduced the expression of *nos2, il1b*, and *tnfa* genes (*n* = 4–5, *p* < 0.05; Student's *t*-test). Moreover, we measured the release of nitric oxide (NO) and IL-1β by microglia cells treated with vehicle or LPS + IFNγ, in the presence or not of CXCL16: as shown in Figures 1F,G, the release of NO (*n* = 7, *p* < 0.05; One-way ANOVA followed by Holm–Sidak *post-hoc* test) and IL-1β (*n* = 4, *p* < 0.05; One-way ANOVA followed by Holm–Sidak *post-hoc* test) induced by LPS + IFNγ was significantly reduced by treatment with CXCL16.

The activation state of microglia cells has been often correlated with their shape, although it is not possible to strictly associate a morphology to a specific phenotype (33). We measured the ramification grade of microglia calculating the “form factor,” a parameter taken as 1 for round cells, and correspondingly < 1 when the morphology deviates from the spherical shape. As shown in Figure 1H, in analogy with what previously reported (29), the form factor of cells polarized toward an anti-inflammatory ramified phenotype (IL-4 20 ng/ml, 24 h) was 0.26 ± 0.03, while in cells with an inflammatory phenotype (LPS+IFNy) was 0.66 ± 0.04. The form factor of cells stimulated with CXCL16 (0.37 ± 0.03) or treated with LPS + IFNy + CXCL16 (0.37 ± 0.04) were similar to cells treated with IL-4, and statistically different from those treated with LPS + IFNy (*n* = 30 cells in three different experiments, *p* < 0.05; One-way ANOVA followed by Tukey *post-hoc* test), further confirming that CXCL16 polarizes cells toward an anti-inflammatory phenotype.

### CXCL16 released by glioma promotes microglia polarization toward an anti-inflammatory phenotype *in vitro*

**Figure 2:**
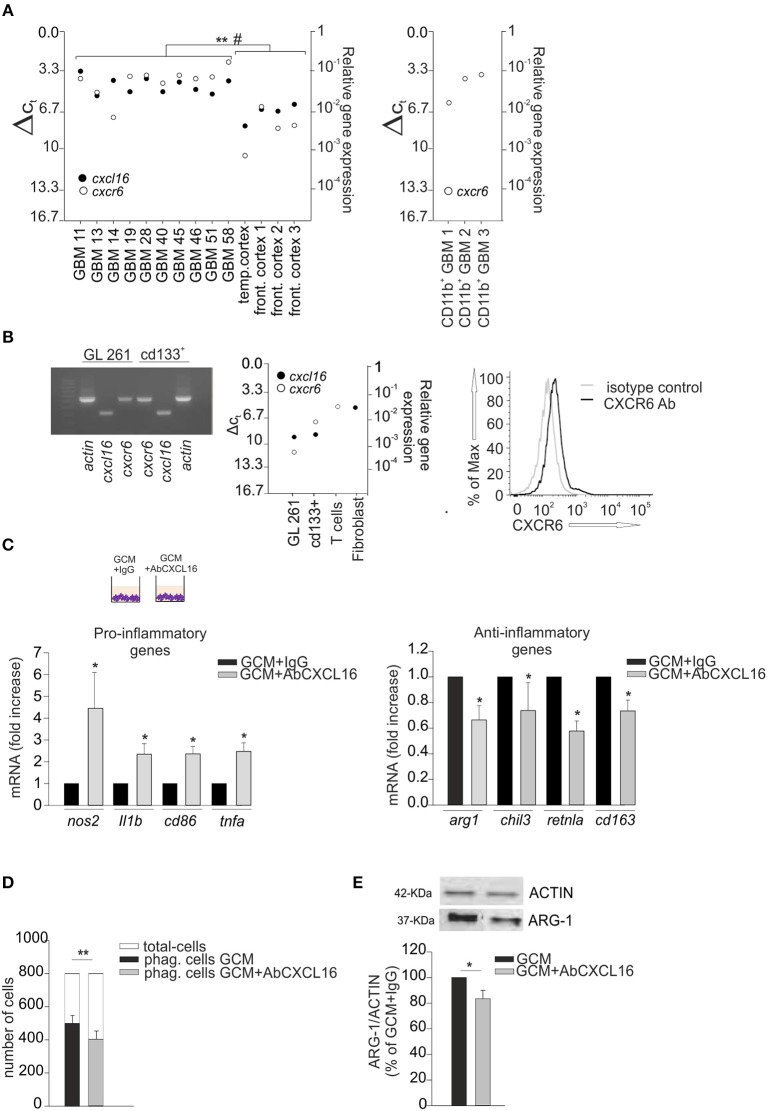
CXCL16 released by glioma cells modulates microglia phenotype. **(A)** Left panel, RT-qPCR for *cxcl16* and *cxcr6* (black and white circles) mRNA expression in human GBM tissues and in human control tissues (temporal or frontal cortex); right panel, qRT-PCR for *cxcr6* mRNA expression in human CD11b^+^ cells (microglia/machrophages) isolated by GBM tissues. Mean values of double measurements from individual patients; ΔCT values = CT gene of interest—CT *gapdh* (housekeeping gene). A ΔCT of 3.33 corresponds to one magnitude lower gene expression compared to *gapd*h; **(B)** Left panel, representative PCR for *cxcl16, cxcr6*, and *actin* mRNAs expression in mouse GL261 cells and mouse GL261 derived stem cells (cd133^+^); right panel RT-qPCR for *cxcl16* and *cxcr6* (black and white circles) mRNAs expression in GL261, cd133^+^ cells, and in mouse T-cells and fibroblasts; CXCR6 surface expression on mouse GL261 cells as evaluated by flow cytometry. Black and gray lines represent CXCR6 staining and isotype control, respectively. **(C)** Expression analysis by RT-qPCR for mRNAs of pro-inflammatory related genes (*nos2, il1b, cd86, tnfa*) (left panel), or anti-inflammatory related genes (a*rg1, chil3, retnla, cd163*) (right panel) in primary *wt* microglia treated with GCM in the presence of AbCXCL16 neutralizing antibody (GCM + AbCXCL16) or control IgG (GCM + IgG). For each gene data are expressed as specific mRNA fold change in GCM + AbCXCL16 treated cells normalized to specific mRNA expression in GCM + IgG treated cells and are the mean (± s.e.m.); **(D)** Phagocytosis of fluorescent beads in microglia cells stimulated with GCM or GCM + AbCXCL16. Data are expressed as number of cells containing 5 or more beads (black/gray bars) within total counted cells (white bars); **(E)** Western-blot analysis of ARG-1 protein expression in microglia cells incubated with GCM or GCM + AbCXCL16. Top representative image; bottom histogram bar of the quantification of ARG-1 expression (data are expressed as ARG-1 signal normalized to ACTIN signal). Statistical analysis: data are expressed as the mean (± s.e.m.) **(A)** **p < 0.001 *cxcl16* expression, ^#^*p* < 0.05 cxcr6 expression, Student's *t*-test; **(C)** *n* = 14, *p < 0.05, Student's *t*-test; for each gene, variability in its expression between control conditions in different experiments never exceeded 10%; **(D)** *n* = 4, **p < 0.001, Student's paired *t-*test; **(E)** *n* = 3, *p < 0.05, Student's *t-*test.

We analyzed the expression of CXCL16 and CXCR6 in human GBM tissues acutely (< 2 h) removed from patients, and in normal cerebral tissues (controls) derived from the temporal and frontal cortex of patients who died for heart failure: RT-qPCR analysis revealed a significant higher expression for *cxcl16* and *cxcr6* mRNAs in GBM, compared to controls (Figure 2A left panel) (*p* < 0.001 and *p* < 0.05, respectively; Student's *t*-test). We also analyzed the expression of *cxcr6* in human CD11b^+^ cells (microglia/macrophages) isolated from GBM tissues, and found a considerable expression of *cxcr6* also in these cells (Figure 2A, right panel). To study the role of CXCL16/CXCR6 in glioma development in a mouse model, we analyzed *cxcl16* and *cxcr6* mRNAs expression in GL261, and in the more aggressive derived glioma stem cells (GL261/cd133^+^ cells): as shown in Figure 2B, both chemokine and its receptor are expressed by GL261 cells, with higher expression of *cxcr6* in GL261/cd133^+^ cells (both by RT- PCR and RT-qPCR analysis). Mouse T-cells and fibroblasts were analyzed as positive controls for *cxcr6* and *cxcl16* mRNAs expression, respectively. Furthermore, we analyzed the expression of CXCR6 membrane protein by flow cytometry, using a mouse CXCR6 PE-conjugated antibody and confirmed the expression of CXCR6 on GL261 cells (Figure 2B, right panel).

It is known that glioma cells secrete soluble factors that contribute to the establishment of a pro-tumor microenvironment switching GAMs toward an anti-inflammatory phenotype (2, 32, 34); thus, considering that microglia cells do express CXCR6, we speculated that CXCL16 released by tumor cells might act as an effector in driving such microglia polarization.

Primary microglia cells were incubated for 24 h with glioma conditioned medium (GCM) in the presence of neutralizing anti-CXCL16 antibody (AbCXCL16) (GCM + AbCXCL16), or control IgG (GCM + IgG), and analyzed for the expression of pro- or anti-inflammatory genes. In the presence of AbCXCL16, microglia increases the expression of *nos2, il1b, cd86, tnfa* (pro-inflammatory genes, Figure 2C, left panel), and decreases the expression of a*rg1, chil3, retnla, cd163* (anti-inflammatory genes, Figure 2C, right panel) compared to cells treated with GCM+IgG (*n* = 14, *p* < 0.05; Student's *t*-test). As control, in each experiment we checked the expression of pro- and anti-inflammatory genes of GCM-incubated microglia (data not shown). These results suggest that CXCL16 in GCM is determinant to promote microglia polarization to establish a pro-tumor/anti-inflammatory microenvironment. To further support these data we analyzed the phagocytizing activity of microglia cells incubated with GCM or GCM + AbCXCL16 (Figure 2D), and the expression of ARG-1 in these cells (Figure 2E): we found a significant reduction in the number of phagocytizing cells (*n* = 4, *p* < 0.001; Student's paired *t*-test) and in the expression of ARG-1 protein (*n* = 3, *p* < 0.05; Student's *t*-test) in cells treated with GCM + AbCXCL16 vs. cells treated with GCM. The presence of soluble CXCL16 in GL261 conditioned medium was also confirmed by ELISA measurement (0.47 ± 0.03 pg/ml).

### CXCR6 expression in glioma recipient mice is determinant for tumor microenvironment

**Figure 3:**
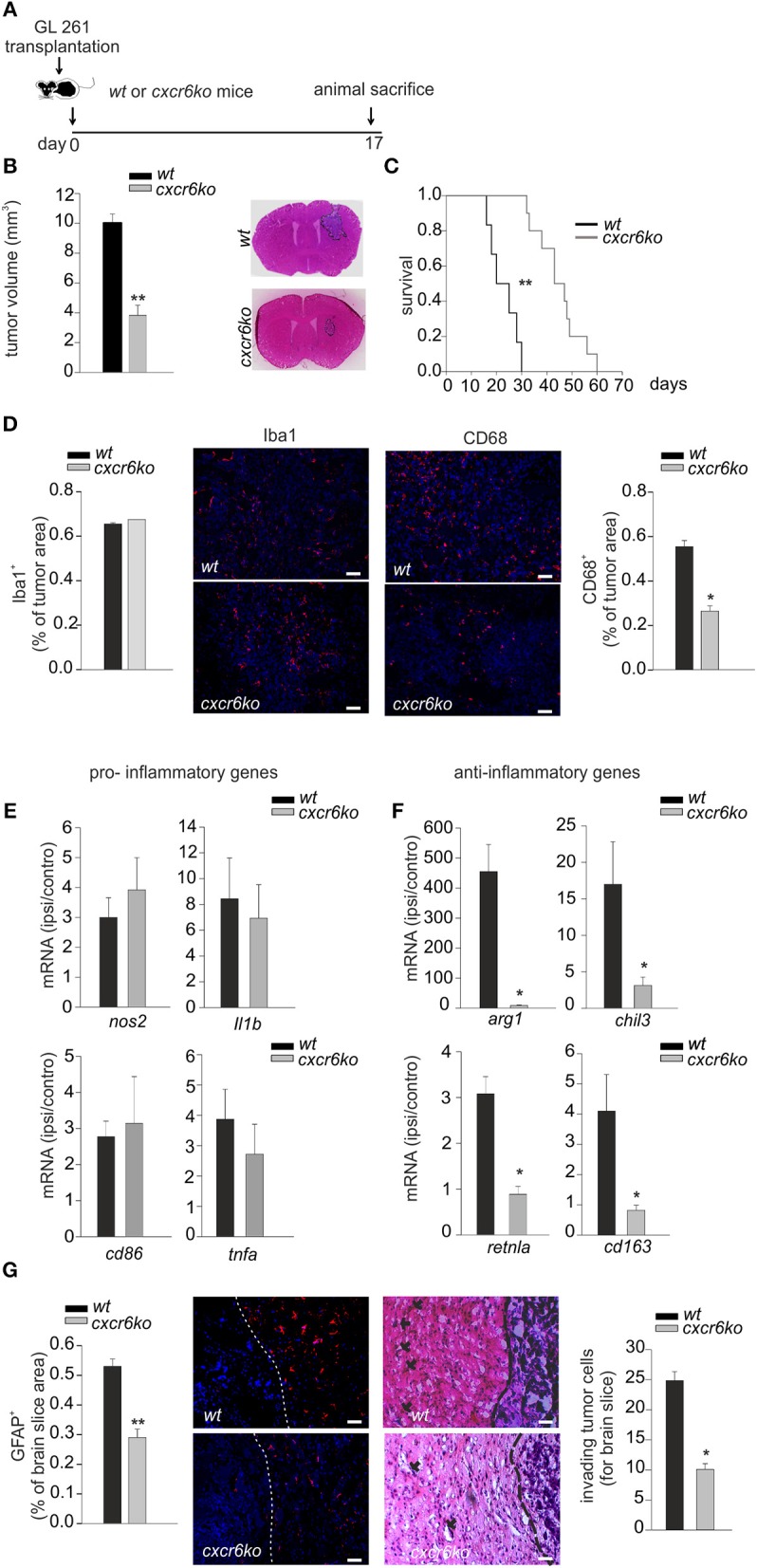
CXCL16/CXCR6 axis is involved in establishing a pro-tumor microenvironment. **(A)** Representative scheme of GL261 transplantation in *wt* and *cxcr6ko* mice; **(B)** bar histogram of the mean (± s.e.m.) of tumor volume in *wt* and *cxcr6ko* mice, and representative hematoxylin-eosin stained coronal brain sections of GL261 bearing mice; **(C)** Kaplan–Meier survival curves of *wt* and *cxcr6ko* GL261 bearing mice; **(D)** Immunofluorescence analysis of Iba1 and CD68 expression in tumor bearing brain slice 17 days after GL261 transplantation in *wt* and *cxcr6ko* mice: (central panels) representative immune-fluorescence images for Iba1^+^, CD68^+^ (red signals), nuclei are evidenced with Hoechst (blue signal), scale bar = 20 μm; (left and right panels): bar histograms representative of the immunofluorescence analysis of Iba1^+^, CD68^+^. Data are expressed as % of Iba^+^ or CD68^+^ staining per tumor area; **(E,F)** RT-qPCR for pro-inflammatory and anti-inflammatory genes in CD11b^+^ cells isolated from the ipsilateral and controlateral brain hemispheres of *wt* and *cxcr6ko* mice transplanted with GL261 cells. Data are expressed as mRNA fold increase in the ipsilateral hemisphere vs. the controlateral hemisphere, normalized for *gapdh* mRNA, and are represented as the mean (± s.e.m.); **(G)** Left, bar histogram representative of immunofluorescence analysis of GFAP^+^ positive cells in tumor bearing brain slice (data are expressed as % of GFAP^+^ area in brain slice), and representative images of GFAP^+^ staining (red signal); right, analysis of glioma cell invasion of surrounding brain tissue in *cxcr6ko* or *wt* mice injected with GL261.Representative coronal brain sections stained with hematoxylin/eosin. Black arrows indicate glioma cells invading the brain parenchyma beyond the main tumor border (dashed line) for more than 150 μm (scale bars, 20 μm) and bar histogram of the number of invading tumor cells 17 days after glioma cell transplantation. Statistical analysis: Data are expressed as the mean number (± s.e.m.) **(B)** *n* = 7–12, **p < 0.001, Student's *t*-test; **(C)** *n* = 6–10, **p < 0.001, long-rank test; **(D)** *n* = 4, *p < 0.05, Student's *t*-test; **(E,F)** *n* = 4–5, *p < 0.05, Student's *t*-test. **(G)** *n* = 3–4, **p < 0.001, *p < 0.05, Student's *t*-test.

Since tumor micro-environment plays an important role in glioma progression, and considering the ability of CXCL16 to promote microglia anti-inflammatory phenotype *in vitro*, we decided to investigate the effect of CXCL16/CXCR6 signaling on tumor micro-environment *in vivo*: we therefore orthotopically implanted GL261 cells into the brain of *wt* and *cxcr6ko* mice. Some animals were used for a survival-analysis, others were sacrificed 17 days after implantation for tumor volume analysis (Figure 3A). As reported in Figure 3B, tumor volume was strongly reduced (62%) in *cxcr6ko* mice compared to *wt* mice (*cxcr6ko*: 3.83 ± 0.67 mm^3^; *wt*: 10.07 ± 0.55 mm^3^; *n* = 7–12, *p* < 0.001; Student's *t*-test). Moreover, survival studies (Kaplan–Meier analysis) revealed that *cxcr6ko* mice survive longer than *wt* mice (*cxcr6ko*: 45 ± 2.9 days; *wt*: 22.8 ± 2.3 days, *n* = 6–10; *p* < 0.001 Log rank test, Figure 3C). These data suggest that the CXCL16/CXCR6 axis plays a key role in establishing a pro-tumoral microenvironment in the brain of glioma-bearing mice. Due to the importance of GAMs in glioma progression, we also investigated Iba1 and CD68 cell immuno-reactivity. As shown in Figure 3D there was no difference in Iba1^+^cells (measured as % of Iba^+^ staining per tumor area) in *wt* and *cxcr6ko* mice (*cxcr6ko*: 0.69 ± 0.02%; *wt*: 0.72 ± 0.04%; *n* = 4, p = 0.56; Student's *t*-test), but there was a strong reduction in CD68^+^cells (measured as % of CD68^+^ staining per tumor area) in *cxcr6ko* mice compared to *wt* mice (53% reduction, *cxcr6ko*: 0.26 ± 0.02%; *wt*: 0.55 ± 0.02%; *n* = 4, *p* < 0.05; Student's *t*-test), indicating that although there was no difference in the recruitment of total GAMs in tumor mass, they were differently activated in *cxcr6ko* mice.

To confirm a role of CXCL16 in driving GAMs toward a pro-tumor phenotype *in vivo*, we implanted GL261 cells into the brain of *wt* and *cxcr6ko* mice and, after 17 days, CD11b^+^ cells were isolated from the ipsi- and contra-lateral brain hemispheres of each mice and analyzed by RT-qPCR. Data reported in Figure 3E show no significant differences in the expression levels of pro-inflammatory genes (*n* = 4–5, *p* > 0.05; Student's *t*-test); instead we found a significant reduction in the expression of anti-inflammatory genes such as a*rg1, chil3, retnla, cd163* in CD11b^+^ cells from *cxcr6ko* mice (Figure 3F; *n* = 4–5, *p* < 0.05; Student's *t*-test). In order to look at differences in the brain tumor microenvironment in the two genotypes, we also analyzed astrocytic activation and tumor cells invasion in the surrounding brain tissue.

As reported in Figure 3G (left), the brain of glioma-bearing *cxcr6ko* mice showed reduced astrogliosis (measured as % of GFAP^+^ area in brain slice) compared to *wt* mice (46% reduction, 0.53 ± 0.03% in *wt*, 0.29 ± 0.03% in *cxcr6ko*; *n* = 4, *p* < 0.001; Student's *t*-test). In addition, as revealed by the analysis of the number of glioma cells protruding more than 150 μm from the main tumor mass, *cxcr6ko* mice presented a reduction in the number of glioma cells invading the brain parenchyma (10.1 ± 1.0 cells for brain slice) compared to *wt* mice (24.8 ± 1.5 cells for brain slice) (*n* = 3, *p* < 0.05; Student's *t*-test), Figure 3G (right).

### Direct effects of CXCL16/CXCR6 axis on glioma cells

**Figure 4:**
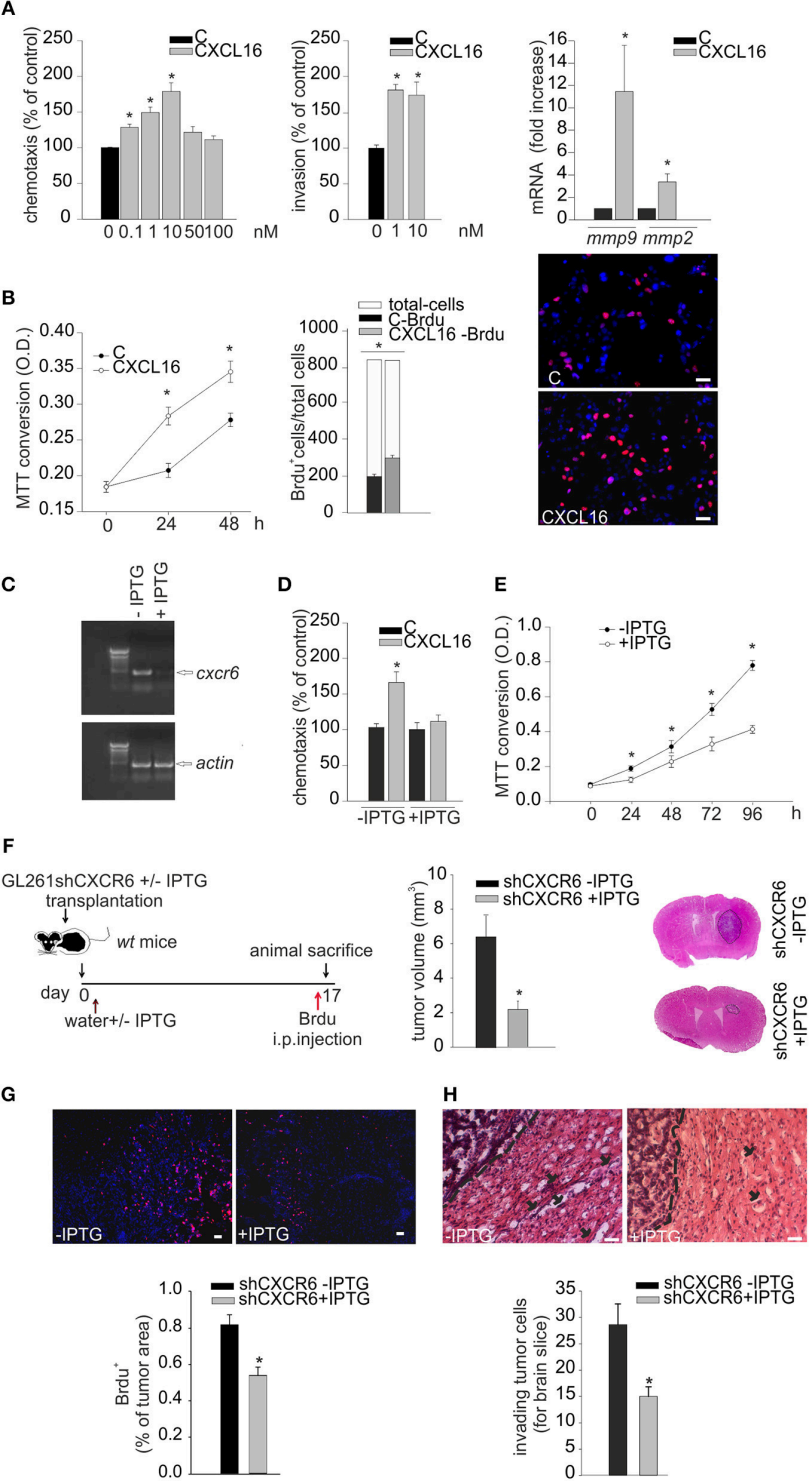
Biological effects of CXCL16/CXCR6 axis on mouse glioma cells. **(A)** Left panel, chemotactic assay (24 h) of GL261 cells in the absence (C) or presence of different doses of CXCL16. Data are expressed as percentage of cell migration toward CXCL16 *vs*. control. Central panel, invasion assay of GL261 cells in the absence (C) or presence of different CXCL16 concentrations (1, 10 nM, 48 h). Data are expressed as percentage of cell invasion in CXCL16 stimulated cells vs. control. Right panel, expression analysis of *mmp2, mmp*9 metalloproteinases mRNAs by RT-qPCR in GL261 cells not stimulated (vehicle), or stimulated with CXCL16 (10 nM, 24 h). For each gene data are expressed as specific mRNA fold increase in CXCL16 treated cells normalized to specific mRNA expression in vehicle; **(B)** Left panel, growth curve (0, 24, 48 h) of GL261 cells unstimulaed (C) or stimulated with CXCL16 (10 nM); data are expressed as MTT conversion optical density; middle panel, BrdU incorporation assay on GL261 stimulated with CXCL16 (10 nM, 4 h), or not (C). Data are expressed as number of BrdU^+^ cells (black or gray bars) within total counted cells (white bar); Right panel, representative images (scale bar = 20 μm) of BrdU incorporation in GL261 cells unstimulated or stimulated with CXCL16 (BrdU, red signal; Hoechst, blue signal); **(C)** RT-PCR for *cxcr6* and *actin* mRNAs expression in GL261 shCXCR6 cells induced or not with IPTG (+/–IPTG); **(D)** Chemotactic assay of GL261shCXCR6 induced or not with IPTG, not stimulated (C) and stimulated with CXCL16 (10 nM; 24 h); data are expressed as percentage of cell migration toward CXCL16 vs. control; **(E)** Growth curve (0, 24, 48, 72, 96 h) of GL261shCXCR6 cells induced or not with IPTG (+/–IPTG); data are expressed as MTT conversion optical density; **(F)** (left) representative scheme of GL261shCXCR6 cells transplantation in *wt* mice; (right) bar histogram of the mean (± s.e.m.) of tumor volume in shCXCR6-IPTG or shCXCR6+IPTG treated mice, and representative hematoxylin-eosin stained coronal brain sections of glioma bearing mice; **(G)** BrdU proliferation analysis in mice transplanted with GL261shCXCR6 cells induced or not with IPTG. (Top) representative images (scale bar = 20 μm) of proliferating BrdU+ cells (red) within tumor area; (bottom) bar histograms of immunofluorescence analysis of BrdU^+^ cells; data are expressed as % of BrdU^+^ staining per tumor area. **(H)** Analysis of glioma cells invasion of surrounding brain tissue in mice injected with GL261shCXCR6 treated or not with IPTG. (Top), Representative coronal brain sections stained with hematoxylin/eosin. Black arrows indicate glioma cells invading the brain parenchyma beyond the main tumor border (dashed line) for more than 150 μm. Bottom, bar histogram of the number of glioma invading cells. Statistical analysis: Data are expressed as the mean number (± s.e.m.) **(A)** left panel *n* = 3 in triplicate, *p < 0.05, one-way ANOVA followed by Holm–Sidak *post-hoc* test; right panel *n* = 3, *p < 0.05, one-way ANOVA followed by Holm–Sidak *post-hoc* test; **(B)** left panel *n* = 3 six-replicates, *p < 0.05, one-Way ANOVA followed by Student-Newman-Keuls Method; central panel *n* = 3, *p < 0.05, Student's *t*-test; **(D)** *n* = 4 in duplicate, *p < 0.05, two-way ANOVA followed by Holm–Sidak *post-hoc* test; **(E)** *n* = 4 in quadruplicates, *p < 0.05, one-Way ANOVA followed by Student-Newman-Keuls Method; **(F)** *n* = 5, *p < 0.05, Student's *t*-test); **(G)** *n* = 3, *p < 0.05, Student's *t*-test; **(H)** *n* = 3–4, *p < 0.05, Student's *t*-test.

We investigated the direct effects of CXCR6 stimulation on GL261 cells: at this aim cells were stimulated with CXCL16 and analyzed for migration in the Boyden chamber assay. Data reported in Figure 4A (left panel) demonstrate that the chemotactic index of GL261 increased with CXCL16 dose, starting at 0.1 nM CXCL16, with maximal effect at 10 nM (*n* = 3 experiments in triplicate, *p* < 0.05; One-way ANOVA followed by Holm–Sidak *post-hoc* test). *In vitro* matrigel invasion assay with GL261 shows significant increase of cell invasion upon CXCL16 stimulation (Figure 4A, central panel; *n* = 3, *p* < 0.05; One-way ANOVA followed by Holm–Sidak *post-hoc* test). In accordance with this effect, we also found that CXCL16 stimulation increased the expression level of the matrix metalloproteinases *mmp9* and *mmp2* (Figure 4A right panel; *n* = 6, *p* < 0.05; Student's *t*-test), whose activity is reported to be involved with the invasion ability of glioma cells (35). To investigate whether CXCL16 might directly promote glioma cell proliferation, in analogy with CXCL12 (20), we analyzed GL261 proliferation upon stimulation with CXCL16. Figure 4B shows that CXCL16 (10 nM) significantly increased GL261 cell number after 24 and 48 h, as revealed by MTT analysis (left panel; *n* = 3 experiments in six-replicates, *p* < 0.05; One way ANOVA followed by Student-Newman-Keuls Method). Similar results were obtained measuring BrdU incorporation in GL261 cells: CXCL16 administration (10 nM, 4 h) increased the number of proliferating BrdU^+^ cells compared to vehicle (C) stimulated cells (middle and right panels; *n* = 3, *p* < 0.05; Student's *t*-test).

### CXCR6 silencing in GL261 reduces tumor migration and proliferation *in vitro* and *in vivo*

To confirm the role of CXCR6 activation on glioma cells, GL261 cells were engineered for CXCR6 silencing, using an IPTG-inducible shCXCR6 construct. As shown in Figure 4C, we selected a GL261shCXCR6-inducible cell clone that, after 10 days of treatment with IPTG, presented a strong reduction in CXCR6 mRNA expression compared to control cells (not treated with IPTG). To further confirm CXCR6 silencing in the selected clone, we performed chemotaxis experiments toward CXCL16: Figure 4D shows that shCXCR6 cells (+IPTG) did not respond to CXCL16 (*n* = 4 experiments in duplicate, *p* < 0.05; Two- way ANOVA followed by Holm–Sidak *post-hoc* test). Since CXCL16 is present in GCM and CXCL16 stimulation increases GL261 proliferation (Figure 4B), we speculated that basal cell proliferation might be altered in shCXCR6 cells: as reported in Figure 4E, cell proliferation measured at 24, 48, 72, 96 h was reduced in IPTG induced GL261shCXCR6 cells (+IPTG) compared to GL261shCXCR6 not treated cells (-IPTG) (*n* = 4 experiments in quadruplicate, *p* < 0.05;One way ANOVA followed by Student-Newman-Keuls Method).

To confirm a role for CXCR6 in glioma development also *in vivo*, we orthotopically implanted GL261shCXCR6 cells, silenced or not with IPTG (+/-IPTG), into the brain of *wt* mice. Tumor-bearing mice were supplied with drinking water with or without IPTG, respectively, and, 17 days after implantation, were sacrificed for tumor volume analysis (Figure 4F). Mice injected with tumor cells silenced for CXCR6 revealed a significant reduction (67%) in tumor volume compared to mice injected with GL261 expressing CXCR6 (2.19 ± 0.47 mm^3^ shCXCR6 + IPTG mice vs. 6.54 ± 1.26 mm^3^ shCXCR6–IPTG) (*n* = 5, *p* < 0.05; Student's *t*-test). These mice were also i.p. injected with BrdU 4 h before sacrifice, in order to analyze tumor cell proliferation *in vivo*: as shown in Figure 4G, shCXCR6+ IPTG cells revealed a significant reduction in BrdU incorporation (0.52 ± 0.08% of tumor area) compared to control cells (0.81 ± 0.05% of tumor area) (*n* = 3, *p* < 0.05; Student's *t*-test). Moreover, as reported in Figure 4H, in mice injected with tumor cells silenced for CXCR6 there was a reduced number of glioma cells that migrate and invade the surrounding brain tissue (15.00 ± 1.8 cells for brain slice) compared to mice injected with cells not silenced (28.6 ± 3.9 cells for brain slice) (*n* = 3–4, *p* < 0.05; Student's *t*-test).

### CXCL16 released from glioma plays a role in tumor development *in vivo*

**Figure 5:**
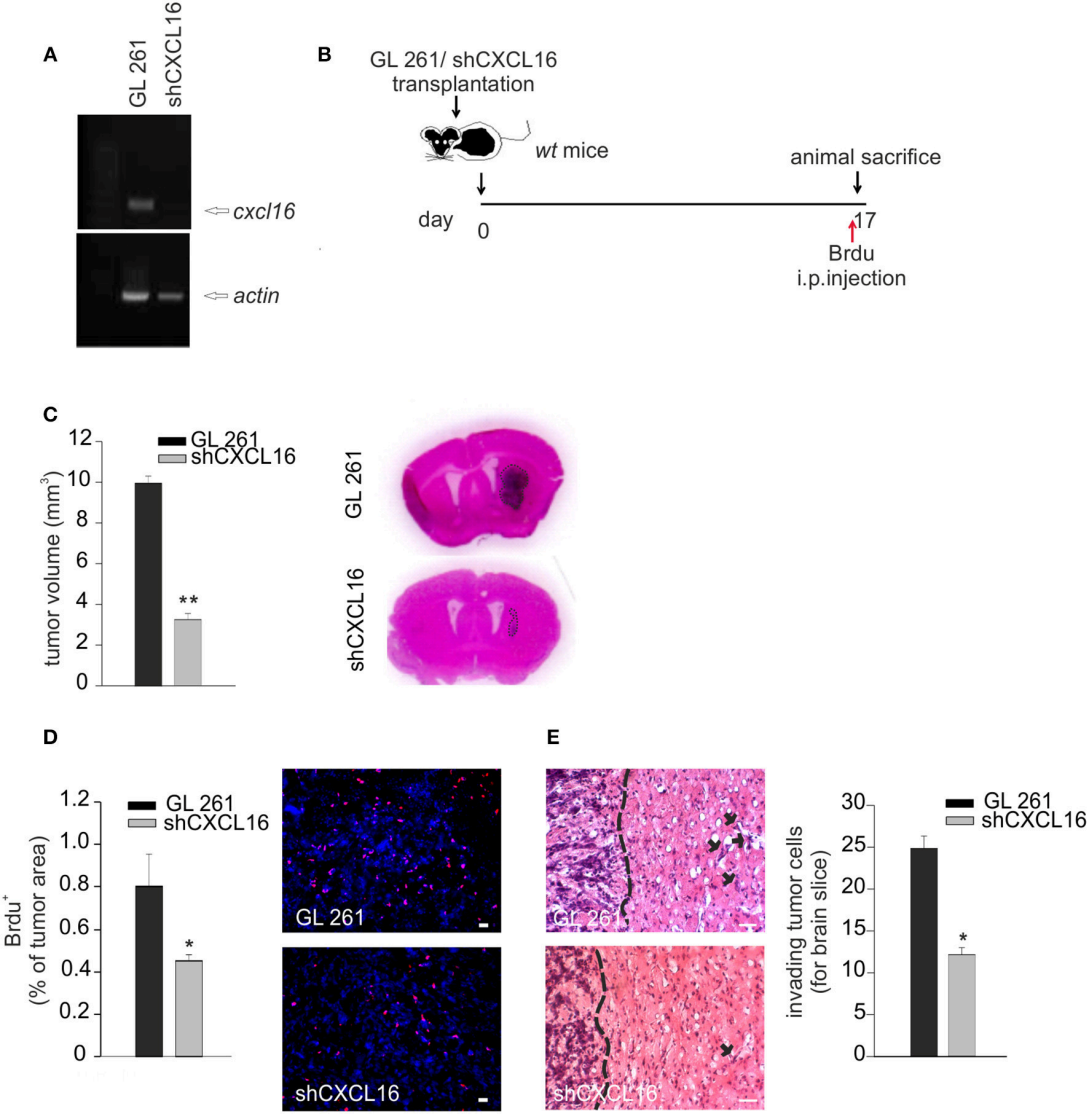
CXCL16 released by glioma cells acts promoting tumor proliferation *in vivo*. **(A)** RT-PCR for *cxcl16* and *actin* mRNAs expression in GL261 and shCXCL16 cells; **(B)** representative scheme of GL261 and shCXCL16 cells transplantation in *wt* mice; **(C)** bar histogram of tumor volume in GL261 or shCXCL16 cells bearing mice, and representative hematoxylin-eosin stained coronal brain sections; **(D)** BrdU proliferation analysis in mice bearing GL261 or shCXCL16 cells. Left panel, bar histograms of immunofluorescence analysis of BrdU^+^ cells; data are expressed as % of BrdU^+^ staining per tumor area; right panel, representative images (scale bar = 20 μm) of proliferating BrdU+ cells (red) within tumor area; **(E)** Analysis of glioma cells invasion of surrounding brain tissue in mice injected with GL261shCXCL16 or GL261. Representative coronal brain sections stained with hematoxylin/eosin. Black arrows indicate glioma cells invading the brain parenchyma beyond the main tumor border (dashed line) for more than 150 μm. Right, bar histogram of the number of glioma invading cells. Statistical analysis: Data are expressed as the mean (± s.e.m.) **(C,D)** *n* = 4, *p < 0.05, **p < 0.001, Student's *t*-test; **(E)** *n* = 3, *p < 0.05, Student's *t*-test.

To prove a role of CXCL16 released from glioma cells in tumor progression, GL261 cells were engineered for constitutive CXCL16 silencing, using shCXCL16 construct. As shown in Figure 5A, we selected a shCXCL16 cell clone with 80% reduction in CXCL16 mRNA expression compared to GL261 cells. GL261 or shCXCL16 cells were implanted into the brain of adult *wt* mice and 17 days after implantation mice were sacrificed for tumor volume analysis (Figure 5B). As reported in Figure 5C, mice injected with shCXCL16 cells revealed a significant reduction (67%) in tumor volume compared to mice injected with GL261 (3.3 ± 0.40 mm^3^ shCXCL16 vs. 9.6 ± 0.67 mm^3^ GL261) (*n* = 4; *p* < 0.001; Student's *t*-test). Glioma-bearing mice were also injected with BrdU 4h before animal sacrifice: analysis of BrdU incorporation in tumor cells (Figure 5D) revealed a significant reduction in BrdU^+^ cells in mice implanted with shCXCL16 cells (0.45 ± 0.02% of tumor area), compared to mice implanted with GL261 cells (0.8 ± 0.15% of tumor area) (*n* = 3; *p* < 0.05; Student's *t*-test). In addition (Figure 5E) we found that mice injected with tumor cells silenced for CXCL16 presented a reduced number of glioma cells that migrate and invade the surrounding brain tissue (12.16 ± 0.82 cells for brain slice) compared to mice injected with GL261 (24.85 ± 1.49 cells for brain slice) (*n* = 3, *p* < 0.05; Student's *t*-test). To exclude that differences observed in *vivo* between tumor volume in GL261 and shCXCL16-GL261 bearing mice might be due to clonal differences between cells rather than to the CXCL16 silencing, we checked for the expression of markers involved in tumor immune recognition (specifically MHC class I, CD1 and PD-L1) as well as tumor cell migration and invasion (CD44). Flow cytometry analysis revealed no differences in the expression of all these markers (Supplementary Figure 1). All these data confirm that CXCL16 released by glioma cells concurs to tumor progression, and promotes tumor cell proliferation.

### Effects of CXCL16/CXCR6 axis in patient's derived GBM cells

**Figure 6:**
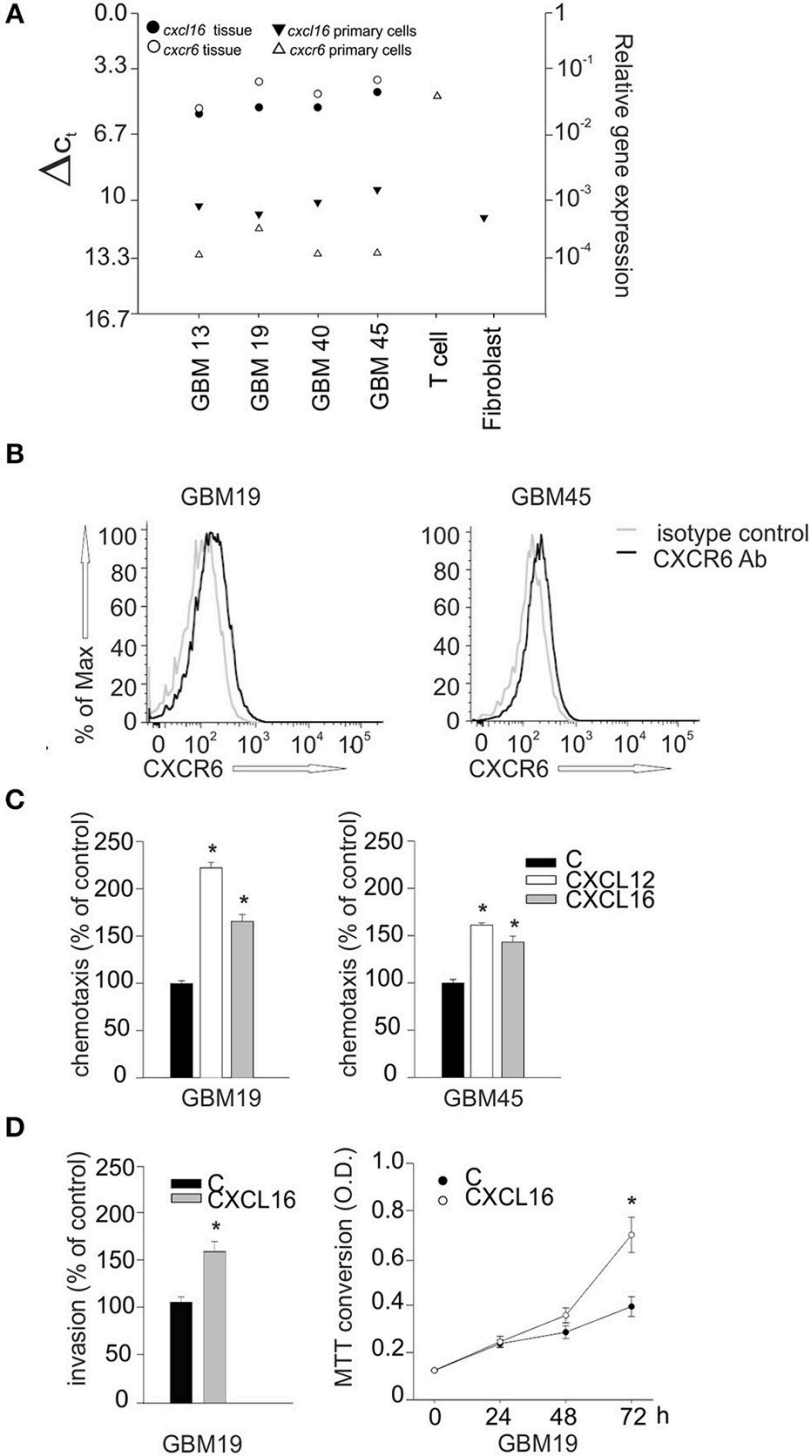
Biological effects of CXCL16/CXCR6 axis on primary human glioblastoma cells.**(A)** *cxcl16* and *cxcr6* mRNAs expression in human GBM tissues (black and white circles), in human primary GBM cells derived from the same tissues and in human primary T cells and fibroblasts (black and white triangles) determined by RT-qPCR; **(B)** CXCR6 surface expression on primary GBM19 and GBM45 as evaluated by flow cytometry. Black and gray lines represent CXCR6 staining and isotype control, respectively; **(C)** Chemotaxis assay of human primary glioblastoma cells: GBM19 and GBM45, toward CXCL16 (10 nM, 4 h), CXCL12 (50 nM, 4 h) and vehicle (C). Data are expressed as percentage of cell migration vs. control; **(D)** Left panel, matrigel invasion assay on human GBM19, toward CXCL16 (10 nm, 24 h) and vehicle (C). Data are expressed as percentage of cell invasion vs. control (*n* = 3, *p* < 0.05, Student's *t*-test); right panel, proliferation assay of GBM19 upon stimulation with CXCL16 (10 nM) at different time points (0, 24, 48, and 72 h), data are expressed as MTT conversion optical density. Statistical analysis: Data are expressed as the mean (± s.e.m.) **(C)** *n* = 4, *p < 0.05, one-way ANOVA followed by Holm–Sidak *post-hoc* test; **(D)** left panel *n* = 3, *p < 0.05, Student's *t*-test; right panel *n* = 3 in five replicates, *p < 0.05, Student's *t*-test.

To investigate whether CXCL16/CXCR6 also modulates cell migration, invasion and proliferation in human GBM, we isolated tumor cells from patient's derived biopsies (GBM 13, 19, 40, 45) and analyzed their expression of CXCL16 and CXCR6 by RT-qPCR. We first compared the expression level of *cxcl16* and *cxcr6* mRNAs in whole patient's tissue, and in the corresponding isolated tumor cells. RNAs from primary human T-cells and a fibroblasts were used as positive controls for *cxcr6* and *cxcl16* expression, respectively. All the examined tissues express high levels of *cxcl16* and *cxcr6*, compared to normal brain tissues (see Figure 2A); however, the corresponding primary cells, even if cultured for only few passages (from 1 to 3), showed a strong reduction in their expression level (Figure 6A). This reduction could be due to a culture-dependent variation in the expression level in tumor cells, but we cannot exclude that the differences observed are due to the selection of specific cell subpopulation (or the elimination of infiltrating cells in the tumor tissue) during the cell culture procedures. By flow cytometry analysis, using specific human CXCR6 PE-conjugated antibody, we confirmed the expression of membrane CXCR6 protein in primary GBM19 and GBM45 cells (Figure 6B). As reported in Figure 6C, primary GBM19 and GBM45, cells responded to CXCL16 stimulation (10 nM, 4 h) increasing their chemotactic index compared to unstimulated cells (control), thus suggesting the expression of functional CXCR6 (*n* = 4, *p* < 0.05; One way ANOVA followed by Holm–Sidak *post-hoc* test). Since it is known that CXCL12 is able to induce migration of GBM cells (36) and GBM19 and 45 cells do express CXCR4 (data not shown), CXCL12 stimulation was used as positive control of migratory activity.

GBM19 cells were also used to investigate CXCL16-induced cell invasion trough matrigel substrate (CXCL16 10 nM, 24 h) and cell proliferation (CXCL16 10 nM, 24, 48, 72 h). As shown in Figure 6D, upon stimulation with CXCL16, there was a significant increase in cell invasion (left panel; *n* = 3, *p* < 0.05; Student's *t*-test), and in cell proliferation (right panel; *n* = 3 experiments in five replicates, *p* < 0.05; Student's *t*-test) compared to un-stimulated cells (control). All these data indicate that activation of CXCL16/CXCR6 axis in human primary GBM cells is able to promote tumor cell proliferation, migration and invasion. To further support our data, we used cBioportal Database to look at a possible correlation between patient survival and CXCR6 expression in glioma tumor. We first explored the alteration in *cxcr6* gene in a merged cohort of low grade glioma (LGG) and GBM (TCGA, Cell 2016) and found a significant increase in patient months survival associated with *cxcr6* deletion (median months survival 130.7 vs. 20.6 in normal cases, Log rank Test *P*-value 0.0339); we than looked at *cxcr6* mRNA expression data in a cohort of Glioblastoma (TCGA, Cell 2013) and found a significant decrease in months survival associated with *cxcr6* mRNA overexpression (median months survival 5.2 vs. 14 in normal cases, Log rank Test *P*-value 0.00417), Supplementary Figure 2.

## Discussion

Communication among cells in the brain parenchyma, including neurons, astrocytes and microglia, is determinant to maintain brain homeostasis. The identification of key players in the cellular cross-talk within the brain, and their alterations in pathological conditions, can be useful to develop specific tools to limit brain damage.

In this paper we report for the first time that: (i) CXCL16 drives microglia toward an anti-inflammatory phenotype, able to counteract inflammatory conditions “*in vitro*”; (ii) CXCL16 released by glioma cells drives GAMs polarization toward an anti-inflammatory phenotype which is determinant to promote glioma progression; (iii) CXCL16 released by tumor cells contributes to glioma cell proliferation, migration, and invasion of brain parenchyma.

Recently we reported that CXCL16, acting on astrocytes, drives neuroprotective effects in brain ischemia, counteracting glutamate excitotoxic damage (11, 12). Besides glutamate-excitotoxicity, also neuroinflammation is a common feature to many chronic or acute neurodegenerative disorders, including brain ischemia. Following acute brain damages, microglia cells at the site of injury produce anti-inflammatory cytokines, scavenger receptors, and trophic factors thus promoting restorative processes. However, later on, microglia acquire a pro-inflammatory phenotype releasing pro-inflammatory cytokines, chemokines, and inducible nitric oxide synthase, all involved in the exacerbation of brain damage (37, 38).

In this paper we report that CXCL16 modulates the inflammatory phenotype of microglia *in vitro*: in particular, we found that CXCL16 *per se* is able to drive microglia toward an anti-inflammatory phenotype and that, in the context of an inflammatory microenvironment (LPS and IFNγ), CXCL16 can contrast the acquisition of a pro-inflammatory phenotype. Considering these data, we speculate that, in addition to limit neuronal damage, counteracting excitotoxicity, the release of CXCL16 in response to ischemic insult (12) might also trigger neuroprotection by limiting neuroinflammation.

The same microglia phenotype triggers different effects on brain homeostasis, in a context-dependent way. During the first phase of glioma development, microglia reacts to counteract tumor growth, phagocytizing tumor cells and activating pro-inflammatory T-cell immune response; at later stages, glioma-released factors produce chronic stimuli, contributing to the establishment of a pro-tumoral microenvironment, also switching GAMs toward an anti-inflammatory/pro-tumor phenotype (2, 32, 34, 39). In line with what already reported (40), we found that CXCL16 is over-expressed in human GBM tissues obtained from patients and demonstrated, *in vitro*, that CXCL16 released by glioma cells acts as a mediator for microglia polarization. We report that neutralization of soluble CXCL16 in GCM results in a strong reduction in the expression of anti-inflammatory genes in microglia *(arg-1, chil3, retnla, cd163*), and in a significant increase of pro-inflammatory genes (*nos2, il-1b, cd86, tnfa*), compared to microglia cells exposed to control GCM, suggesting that soluble CXCL16 released by tumor cells promotes microglia pro-tumor phenotype.

Using *cxcr6ko* mice, we confirmed the crucial role of the CXCL16/CXCR6 axis in the establishment of a pro-tumor microenvironment. These mice, transplanted with GL261 cells, have a strong reduction in tumor volume and a significant increase in mice survival when compared to *wt* animals. Moreover, analysis of Iba1 and CD68 immune-reactivity within the tumor mass reveals a different activation state of GAMs in *cxcr6ko* mice, indicating an effect of CXCR6 signaling on GAMs activation. Accordingly, the analysis of CD11b^+^ cells derived from the brain hemispheres of tumor injected mice, confirms the role of CXCL16 signaling in determining GAMs polarization: indeed, the strong up-regulation of anti-inflammatory genes observed in the brain of *wt* animals did not occurred in *cxcr6ko* mice. Other chemokines released by glioma cells, such as CCL2, have been reported to play a role in the recruitment of GAMs within the tumor mass, but do not contribute to their phenotypic changes (18). Thus, CXCL16 is the first chemokine released by glioma cells that has been proven to drive the interplay with GAMs to acquire a phenotype that supports tumor growth.

GBM are characterized by extensive proliferation and dissemination of the tumor cells within the brain that hinders complete surgical resection (41, 42). The high invasion ability of GBM is due to multiple autocrine motility-enhancing signaling systems, and to distinct signals derived from non-tumor infiltrating and stromal cells.

For the first time we demonstrated that CXCL16/CXCR6 axis plays a role in promoting glioma growth, directly acting on tumor cells. Specifically, we demonstrated that: (i) GL261 cells express both CXCL16 and CXCR6; (ii) stimulation with CXCL16 promotes GL261cell migration, invasion, and proliferation; (iii) the silencing of CXCR6 on glioma cells reduces their proliferation rate and migration ability; (iv) *in vivo*, transplantation of CXCR6-silenced GL261 cells in *wt* mice leads to a reduced tumor cell proliferation and infiltration and tumor volume compared to mice injected with not silenced glioma cells.

The absence of CXCR6 on glioma cells, but not on other cells of tumor microenvironment, reduces but does not block tumor development, suggesting that other signals are important for tumor progression, and again confirming that CXCL16/CXCR6 signaling acts also on cells of the tumor microenvironment. The hypothesis that CXCL16 released from tumor cells acts in an autocrine/paracrine way to promote tumor progression is further confirmed by the significant reduction in tumor volume, proliferation, and infiltration in mice bearing glioma cells silenced for CXCL16. We have previously shown that GL261/cd133^+^ cells grafted in mice resulted in a higher tumor volume compared to mice grafted with GL261 (43), we now report that within glioma, GL261/cd133^+^ cells do present a higher *cxcr6* mRNA expression compared to GL261, thus suggesting that autocrine CXCL16 signaling plays also a role in cancer stem cells.

The role of CXCR6 in human glioma cells is controversial: high expression of CXCL16 has been reported in several human GBM cell lines, as well as in human glioma tissues (TCGA database), in contrast to a very low, sometimes almost undetectable, expression of CXCR6 (21). Moreover, by *in situ* hybridization, it has been shown that CXCR6 is expressed in glioma only on a small population of cells that are positive for markers of embryonic or neural stem cells (21). Considering the very low expression level of *cxcr6* in GBM cells, as measured by real-time PCR, authors speculated that CXCR6 could not play a role in glioma cell biology (22). While we confirm that primary human GBM cells from patients express low levels of *cxcr6* and *cxcl16*, we report that the original GBM tissues, acutely dissected from patients, over-express both *cxcl16* and *cxcr6*, compared to human control brain tissues. In spite of the low expression level of *cxcr6*, we demonstrate that the human primary GBM cells do express CXCR6 protein (as revealed by flow cytometry analysis) and respond to CXCL16 stimulation, modulating migration, invasion, and proliferation, thus suggesting an important activity of CXCL16 in glioma cell biology also in humans. According to Hattermann et al. (22), soluble CXCL16 might act with an “inverse signaling” mechanism that is independent by its receptor, and dependent by the transmembrane form of the chemokine expressed by cells; however, we demonstrated that the direct effects of the soluble CXCL16 on GL261 cells, in terms of proliferation, migration and invasion, are prevented when these cells are silenced for the CXCR6 receptor, but still expressing transmembrane CXCL16, both *in vitro* and *in vivo* (Figures 4D–H), highlighting an important activity of CXCR6 at least in these cells. In analogy to what has been recently reported for another GBM-derived molecule, osteopontin, which regulates glioma cell invasiveness and tumor growth (44) and the pro-tumorigenic reprogramming of microglia (45), we demonstrate that soluble CXCL16 released by glioma cells drives GBM growth directly promoting tumor cell proliferation, invasion, and acting on GAMs establishing a pro-tumor microenvironment. We also prove that human infiltrating GAMs do express *cxcr6*, further supporting the idea that also in human, CXCL16 released by tumor cells, might act on these cells promoting a pro-tumor microenvironment.

For the first time we show that CXCL16/CXCR6 axis plays an important role in driving the cross-talk among cells within the brain and microglia, as well as infiltrating macrophages, triggering a phenotype that, depending on environmental cues, can be either neuroprotective or detrimental. These data highlight the potential use of CXCL16 as pharmacological tool to augment the anti-inflammatory cellular response and to restrain inflammatory stimuli. Moreover, since disruption of CXCL16 signaling counteracts glioma progression limiting cell proliferation and migration but also microglia pro-tumor polarization, a multi-target therapy including the use of a CXCR6 antagonist, together with drugs approved by Food and Drug Administration (FDA) and currently used to treat GBM patients (such as Temozolomide or checkpoint inhibitors that target programmed cell death protein 1,PD-1) could be potentially considered in the future.

## Ethics statement

The study was approved by the Policlinico Umberto Primo Ethics Committee and Neuromed Ethics Committee, and the animal experiments were approved by the Italian Ministry of Health in accordance with the guidelines on the ethical use of animals from the European Community Council Directive of September 22, 2010 (2010/63/EU).

## Author contributions

FL and GD designed, performed and analyzed the experiments. FA performed cytofluorimetric analysis. AS and VE surgically resected glioma tissue form GBM patients. CL and FT designed the experiments, analyzed data and wrote the paper.

### Conflict of interest statement

The authors declare that the research was conducted in the absence of any commercial or financial relationships that could be construed as a potential conflict of interest.
