# Supplementary material for: CXCL16/CXCR6 Axis Drives Microglia/Macrophages Phenotype in Physiological Conditions and Plays a Crucial Role in Glioma
Source: Front Immunol. 2018 Nov 27;9:2750. doi: 10.3389/fimmu.2018.02750 (PMC6277753; doi:10.3389/fimmu.2018.02750)

Supplementary Material

CXCL16/CXCR6 axis drives microglia/macrophages phenotype in physiological conditions and plays a crucial role in glioma

Francesca Lepore^1§^, Giuseppina D’Alessandro^2§^, Fabrizio Antonangeli^3^, Antonio Santoro^4^, Vincenzo Esposito^2,4^, Cristina Limatola^2,5*^and Flavia Trettel^5*^

^1^Department of Physiology and Pharmacology, Sapienza University, 00185 Rome Italy

^2^IRCCS Neuromed, Via Atinense 18, 86077 Pozzilli, IS, Italy

^3^Department of Molecular Medicine, Sapienza University, Laboratory affiliated to Istituto Pasteur Italia – Fondazione Cenci Bolognetti, 00161 Rome Italy.

^4^Department of Neurology and Psychiatry, Sapienza University, 00185 Rome, Italy

^5^Department of Physiology and Pharmacology, Sapienza University, Laboratory affiliated to Istituto Pasteur Italia – Fondazione Cenci Bolognetti, 00185 Rome Italy

**^§^**Authors equally contributed to the present work

*** Correspondence:**Dr. Flavia Trettel and Dr. Cristina Limatola
[flavia.trettel@uniroma1.it](mailto:flavia.trettel@uniroma1.it); cristina.limatola@uniroma1.it

**Table 1:** Sequences of the primers used for RT-PCR or RT-qPCR

| **Gene** | **Specie** | **Forward** | **Reverse** |
| --- | --- | --- | --- |
| ***RT-PCR***  *cxcl16*  *cxcr6*  *β-actin* | mouse  mouse  mouse | AAAGAGTGTGGAACTGGTCATG  TCGTTTCATTGTAGTGGTCCAG  GTCACCCACACTGTGCCCAT | AGCTGGTGTGCTAGCTCCAG  CATAAGTTTCCAGACGTTCTTC  ACAGAGTACTTGCCCTCAGGA |

| **Gene** | **Specie** | **Primer** | **Primer** |
| --- | --- | --- | --- |
| ***RT-qPCR***  *nos2*  *Il1-b*  *cd86*  *tnfa*  *arg1*  *chil3*  *retnla*  *cd163*  *cxcl16*  *cxcr6*  *cxcl16*  *cxcr6*  *mmp9*  *mmp2*  *gapdh*  *gapdh* | mouse  mouse  mouse  mouse  mouse  mouse  mouse  mouse  mouse  mouse  human  human  mouse  mouse  mouse  human | ACATCGACCCGTCCACAGTAT  GCAACTGTTCCTGAACTCAACT  AGAACTTACGGAAGCACCCA  GTGGAACTGGCAGAAGAG  CTCCAAGCCAAAGTCCTTAGAG  CAGGTCTGGCAATTCTTCTGAA  CCAATCCAGCTAACTATCCCTCC  GCTAGACGAAGTCATCTGCACTGGG  TCCTTTTCTTGTTGGCGCTG  CCCTTTTGGGCCTATGCAG  GGCCCACCAGAAGCAGCATTTAC  ATGCCATGACCAGCTTTCACT  TAGCTACCTCGAGGGCTTCC  AGGAATCGGGCCTAAAATTG  TCGCTCCCGTAGACAAAATGG  CCCCTTCATTGACCTCAACTAC | CAGAGGGGTAGGCTTGTCTC  ACTTTTTGGGGTCCGTCAACT  GGCAGATATGCAGTCCCATT  CCATAGAACTGATGAGAGG  AGGAGCTGTCATTAGGGACATC  GTCTTGCTCATGTGTGTAAGTGA  ACCCAGTAGCAGTCATCCCA  TCAGCCTCAGAGACATGAACTCGG  CAGCGACACTGCCCCTGGT  ATGCCTCGAAGAGTTTTGCAC  CTGAAGATGCCCCCTCTGAG  TTAAGGCAGGCCCTCAGGTA  GTGGGACACATAGTGGGAGG  TGCTTTTCAGTGTTTTGGTGA  TTGAGGTCAATGAAGGGGTC  GATGACAAGCTTCCCGTTCTC |

**Supplementary Figure 1**: Surface expression of MHC Class I, CD1, PD-L1, and CD44 on GL261 cells, as analyzed by flow cytometry. Gray histogram and black line represent GL261 and shCXCL16, respectively; dotted and dashed lines represent the corresponding isotype controls. The representative experiment here reported highlights absence of modulation after CXCL16 interference for all the investigated markers


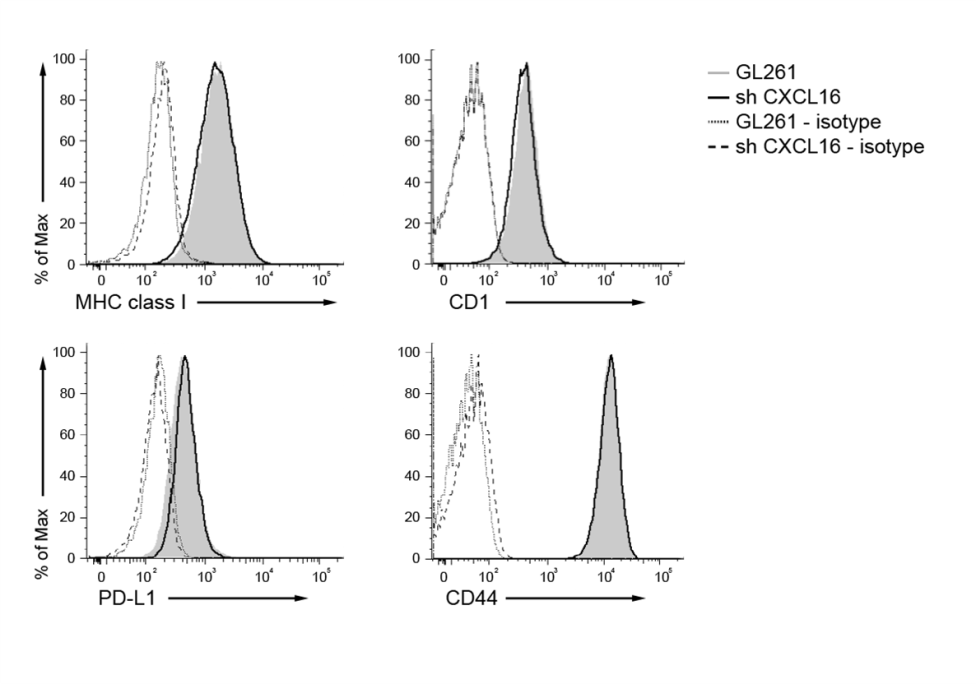


**Supplementary Figure 2**: Patients Survival curves derived from the cBioportal Database. **A**) Data set: merged cohort of LGG and GBM (TCGA, Cell 2016), 1122 samples; **B**) Data set: cohort of Glioblastoma (TCGA, Cell 2013) 585 samples.


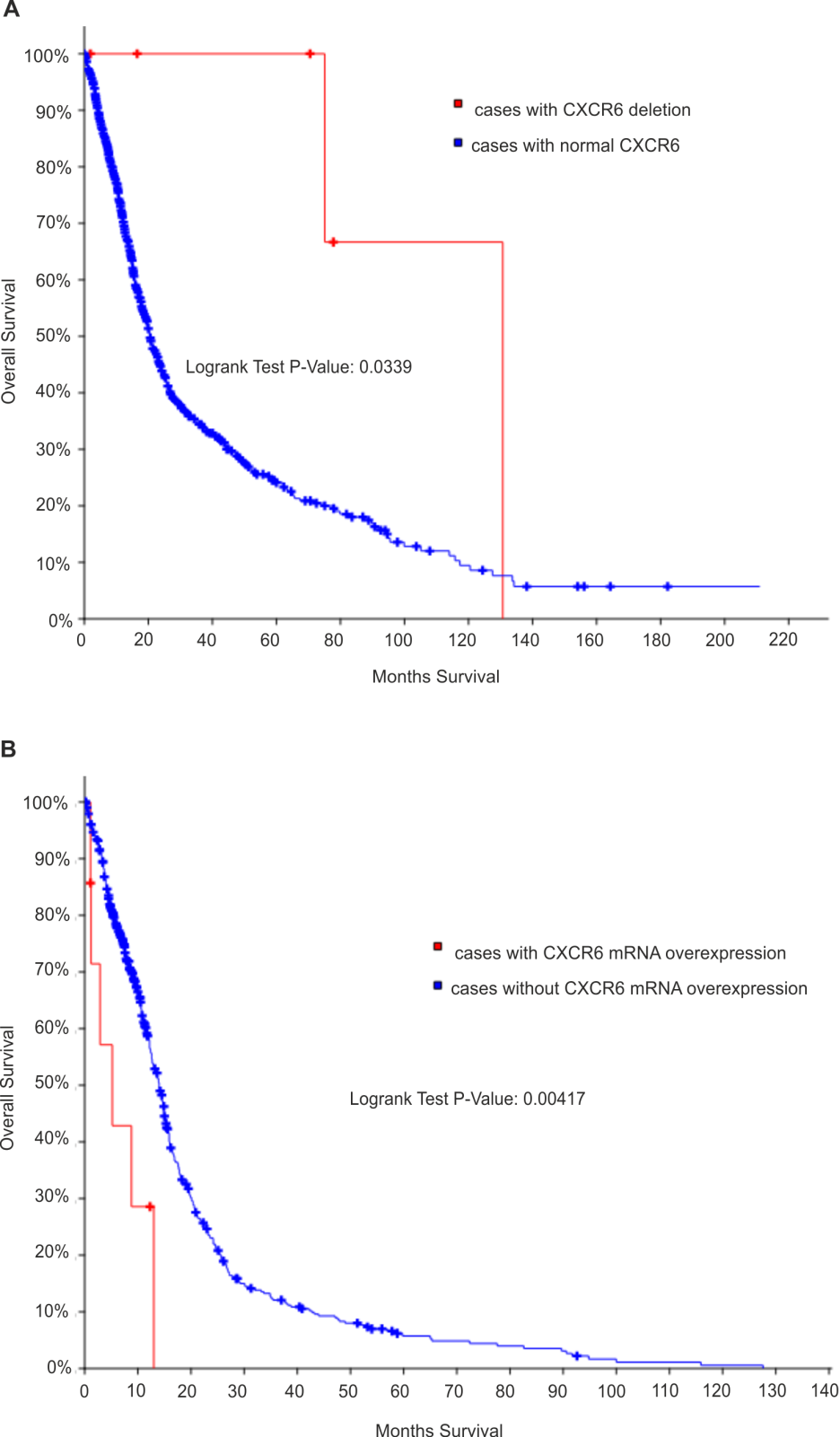

Supplement: Supplementary file 1 [file Data_Sheet_1.docx]
